# Supplementary material for: Spontaneous mutant in threespine stickleback connects endosome trafficking disorders and inflammatory bowel diseases via changes in the gut microbiome
Source: bioRxiv. 2025 Sep 21:2025.09.20.677535. Preprint. [Version 1] doi: 10.1101/2025.09.20.677535 (PMC12458140; doi:10.1101/2025.09.20.677535)
Supplement: 1 [file NIHPP2025.09.20.677535V1-supplement-1.pdf]

bioRxiv preprint doi: https://doi.org/10.1101/2025.09.20.677535; this version posted September 21, 2025. The copyright holder for this preprint (which was not certified by peer review) is the author/funder, who has granted bioRxiv a license to display the preprint in perpetuity. It is made available under aCC-BY 4.0 International license.

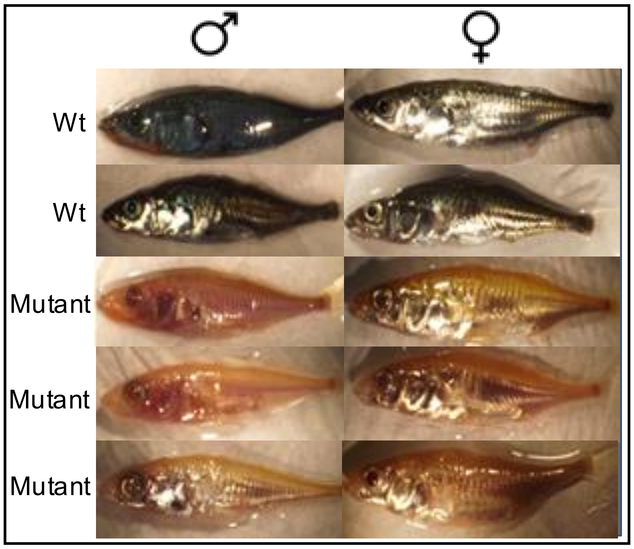

Figure S1. Phenotypic variation by sex of wildtype (Wt) vs Mutant.

bioRxiv preprint doi: https://doi.org/10.1101/2025.09.20.677535; this version posted September 21, 2025. The copyright holder for this preprint (which was not certified by peer review) is the author/funder, who has granted bioRxiv a license to display the preprint in perpetuity. It is made available under aCC-BY 4.0 International license.

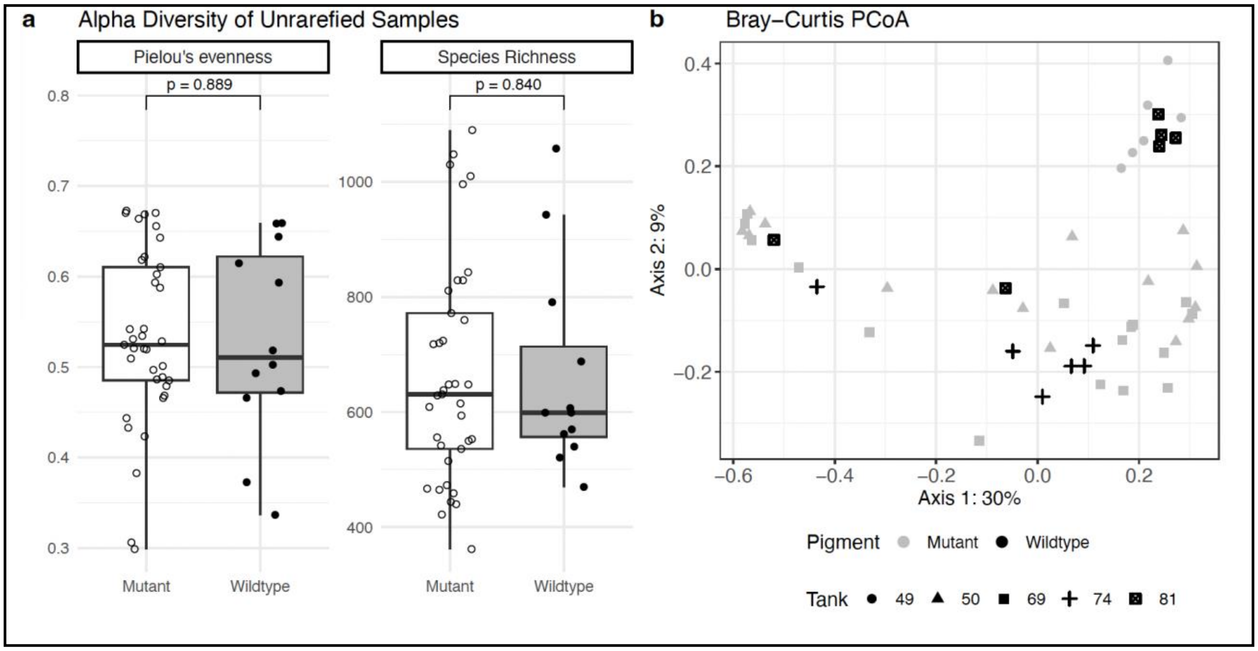

Fig S2: A) Alpha Diversity measured with Pielou's Evenness and Species (ASV) Richness. Differences in alpha diversity between mutant and wildtype stickleback were tested using a linear effects model. No significant differences were detected between mutant and wildtype stickleback. B) Principal Coordinates Analysis of Bray Curtis Dissimilarity between stickleback reveal minor compositional differences in the microbiomes of mutant and wildtype stickleback.
